# Detection of *V. vulnificus* septic shock with ARDS using mNGS

**DOI:** 10.1515/biol-2022-0584

**Published:** 2023-05-18

**Authors:** Tangjun Dan, Haidong Qin, CaiZhi Sun, Hua Shen, Lei Guo

**Affiliations:** Department of Emergency Medicine, Nanjing First Hospital, Nanjing Medical University, Nanjing, China

**Keywords:** *Vibrio vulnificus*, metagenomic sequencing technology, early diagnosis, septic shock

## Abstract

The latest surveillance from the Centers for Disease Control and Prevention shows that the annual incidence of *V. vulnificus* infection is increasing. Unfortunately, in less well-known high-risk groups, this infection is usually excluded from differential diagnosis. Transmitted through wound exposure or ingestion, the mortality rate of foodborne diseases of *V. vulnificus* is the highest of all *V. vulnificus*. *V. vulnificus* is as lethal early diagnosis as Ebola and bubonic plague, so timely treatment is imperative. Sepsis caused by *V. vulnificus* infection mainly exists in the United States and is rarely reported in Southeast Asia. We report a 78-year-old man who went to the local hospital and complained of swelling in his right hand with severe pain. He ate raw salmon 2 days ago and denied other recognized seafood stab or trauma history and other seafood contact history. He was in septic shock at the time of treatment, so we immediately transferred to the emergency intensive care unit and tested for metagenomic next-generation sequencing (mNGS). The diagnosis was confirmed the second day after admission, and eventually he was cured and discharged from the hospital only after medical treatment, thus avoiding the risk of surgical debridement or even amputation. mNGS is helpful for early clinical diagnosis and effective early intervention for etiology, so that patients can get a good prognosis.

## 1 Introduction

*Vibrio vulnificus* is a gram-negative facultative anaerobes distributed in warm brackish water areas. *V. vulnificus* usually infects people through two different entry mechanisms: eating contaminated raw seafood (especially raw oysters) or exposure to seawater or seafood products [1]. People with impaired immune function, especially those with chronic liver disease, alcoholism, and hemochromatosis, have a higher risk of severe infection [2]. The infection routes of *V. vulnificus* are oral infection and wound infection. Its pathophysiology can be divided into three types: (1) primary septicemia; (2) gastrointestinal disease type; and (3) wound infection type. Primary septicemia is caused by oral infection through eating raw seafood and leads to systemic infection of necrotizing fasciitis within 48 h [1,2]. Most cases of *V. vulnificus* infection show primary septicemia, with a mortality rate of more than 50%, and more than half of the cases die within 3 days [3]. Currently, with the development of molecular biology, mNGS provides an alternative approach to assist in early clinical diagnosis with all microbes in a sample identified [4–6]. In this case, the pathogen detection from serum and blisters was performed based on mNGS platform, and this suspected diagnosis was confirmed by quantitative polymerase chain reaction (qPCR) [4]. Their results are usually expressed in terms of relative abundance and total coverage rate. Total coverage rate refers to the proportion of detected sequences to the whole genome sequence, which can be interpreted as how many regions of the whole genome are detected and is usually expressed as a percentage. Relative abundance can refer to both the number of sequences detected for a particular species or genus of microorganism as a proportion of the overall number of sequences detected in the specimen to be tested. Relative abundance greater than 50% is of greater diagnostic significance to the clinic [4].

### 1.1 Case presentation

**Figure 1:**
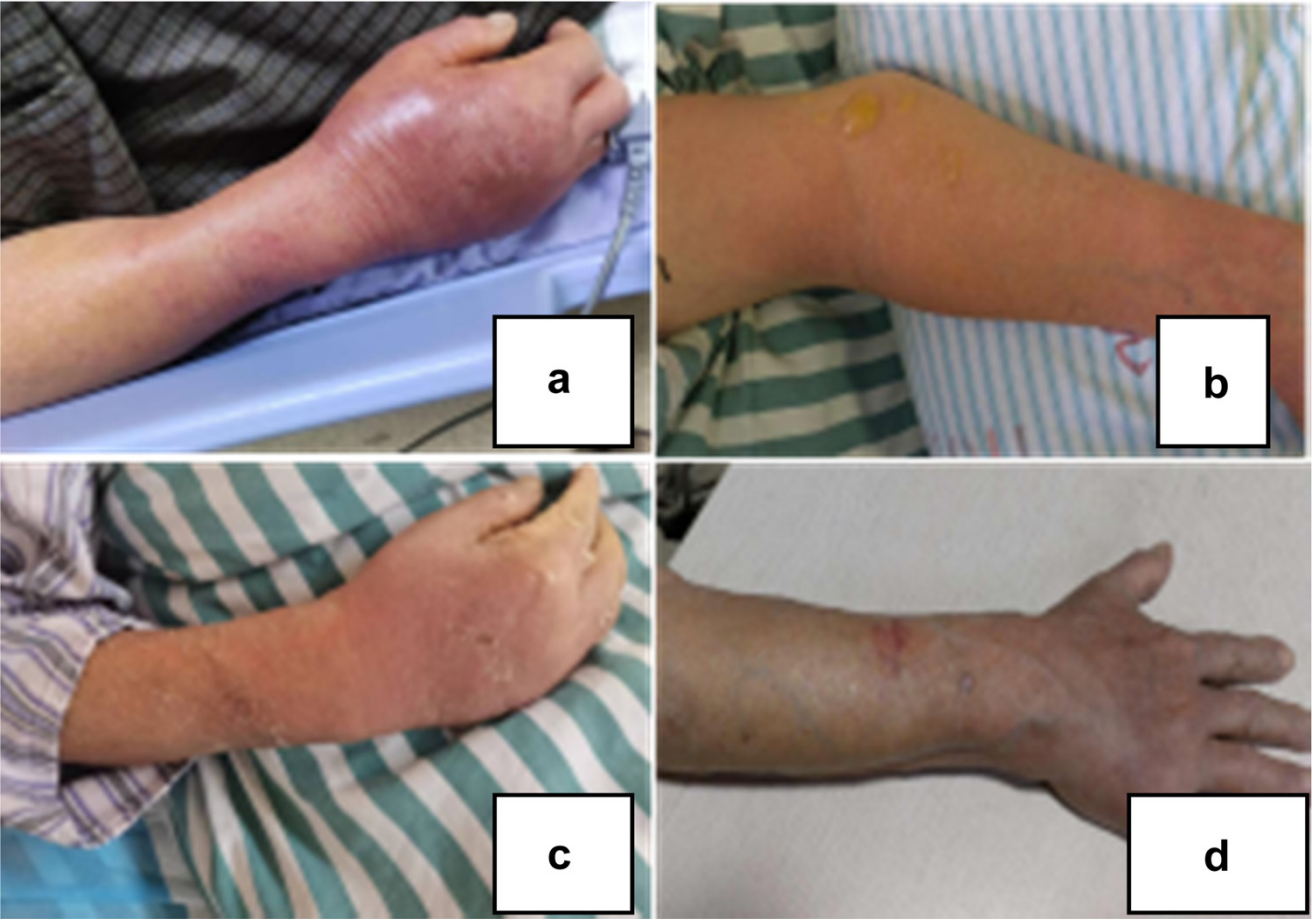
*V. vulnificus* infection in the right forearm. (a) On the first day of admission, the right forearm was swollen and tenderness. (b) On the second day of admission, the scope was enlarged and tension blisters. (c) Swelling and blisters disappear when transferred out of ICU. (d) One month after discharge, the right forearm was recovered as before.

On May 29, 2022, at 1:00 a.m., a 78-year-old male presented to the emergency room with swelling in his right hand, acute discomfort, and chills. His lengthy medical history includes the following: “coronary heart disease,” “hypertension,” and “cardiac insufficiency” in the past, “right kidney transplantation” for 15 years, and “Medrol, tacrolimus” treatment over an extended period of time. He claimed that 2 days previously, at noon, he had eaten raw salmon, and by 7 o’clock that evening, his right upper leg was itchy, red, and swollen. He did not take his body temperature at home, but he was aware that he had previously been exposed to seafood and had suffered no stab wounds. The family members who dined with them did not have any symptoms. Later, the swelling gradually spread and became painful; half a day before admission the patient began to develop asthma and dyspnea, which gradually worsened; physical examination into the emergency room: body temperature 101.3 degrees Fahrenheit, SPO290%, blood pressure 78/54 mmHg (1 mmHg = 0.133 kPa), blurred consciousness, no stab wounds or injuries, high skin temperature on the right forearm and back of the hand, sunken redness and swelling, obvious tenderness (Figure 1a), GCS score 12, qSOFA score 3. Laboratory examination included blood routine: white blood cell count 15.89 × 10^9^/l and neutrophil ratio 90%; emergency biochemistry: PCT 30.359 ng/ml and creatinine 130.30 μmol/l; and blood gas analysis: FiO_2_ 33%, pH 7.48, PaO_2_ 51 mmHg, and lactate 2.2 mmol/L SPO_2_ 88%. Considering septic shock, with acute respiratory distress syndrome (ARDS) the emergency physician was given active fluid replacement and *m*-hydroxylamine to maintain blood pressure. Considering the critical condition and rapid progress, the emergency physician was transferred to the intensive care unit at 2:00 on the same day for further treatment.

**Figure 2:**
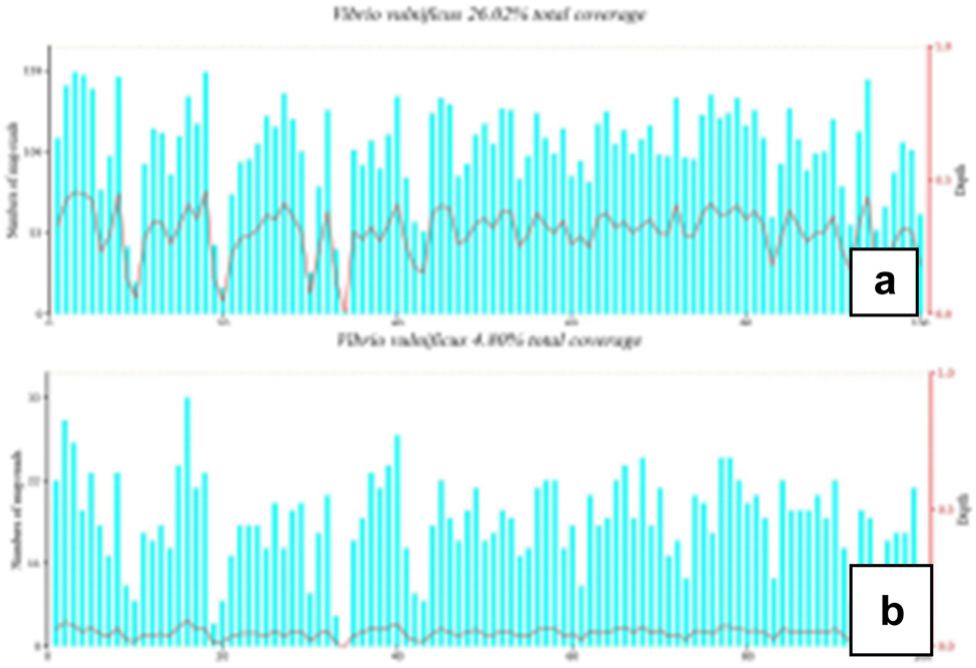
mNGS total sequence coverage of *V. vulnificus*. (a) The total coverage rate of *V. vulnificus* in blood samples is 4.80%. (b) The total coverage rate of *V. vulnificus* in secretion was 26.02%.


After entering the intensive care unit, patients were treated with high flow ventilation, imipenem cilastatin combined with linezolid, *m*-hydroxylamine, fluid replacement, and volume expansion. Blood samples and secretions from infected sites were collected for mNGS and routine microbial culture. The specimens were sent to the Nanjing Shihe Gene Biotechnology company for testing. On the second day after admission, the focus of infection spread to the proximal end of the heart, and tension blisters appeared (Figure 1b). There was no indication of incision in surgical consultation. mNGS results on the second day of admission: the sequence number of *V. vulnificus* in secretion samples was 42,501, the relative abundance was 89.88%, the total coverage rate was 26.02%, and the sequence number, relative abundance, and total coverage rate of *V. vulnificus* in blood samples were 6,523, 52.07%, and 4.80% respectively (Figure 2). The culture of blood and secretion were negative on the fourth day after admission. Clear diagnosis was as follows: septic shock; right forearm *V. vulnificus* infection; *V. vulnificus* septicemia; multiple organ dysfunction syndrome (consciousness, circulation, respiration, kidney); and after renal transplantation. The anti-infective regimen was adjusted to cefoperazone sodium and sulbactam sodium combined with omocycline. Patients were transferred to intensive care unit (ICU) on the ninth day (Figure 1c) and recovered well 1 month after discharge (Figure 1d).

**Informed consent:** Informed consent has been obtained from all individuals included in this study.
**Ethical approval:** The research related to human use has been complied with all the relevant national regulations, institutional policies, and in accordance with the tenets of the Helsinki Declaration and has been approved by the authors’ institutional review board or equivalent committee.

## 2 Discussion

*Vibrio* infections have been linked to global warming from large data studies [7], occurring mainly in the United States [8], but also in southern Europe and Asia [9–11]. In recent years, *V. vulnificus* infections, which are opportunistically pathogenic and have a perennial distribution with a gradual increase, have been ranked as one of the most dangerous bacteria in the southeastern coastal region of China [7,8]. Risk factors include age >60 years, immunosuppression, and chronic kidney disease [3]. Skin infection can be manifested as severe pain, swelling, and tension blisters at the distal end of the limbs, which can quickly spread to the entire limb and even the torso. In this case, the patient has the following characteristics: advanced age, summer onset, history of raw seafood, long-term use of immunosuppressants after renal transplantation, limb swelling and pain, skin blisters after infection, rapid development into septic shock, and multiple organ dysfunction syndrome; so it accords with the characteristics of *V. vulnificus* sepsis.

*V. vulnificus* can be isolated from blood, exudate, blisters, and cerebrospinal fluid, which is the gold standard of clinical diagnosis. Blood should be sent and wound culture should be given at the early stage of infection for rapid diagnosis [12], but many patients have been treated with antibiotics before admission [10,13,14], which may increase the negative conversion rate of culture and complicate diagnosis [15]. The mNGS technology developed in recent years can detect and compare all nucleic acid sequences in samples without specific amplification and report the results within 24 h. It is especially suitable for the diagnosis of acute and critical diseases and difficult infections [16]. In patients treated with antibiotics before admission, skin lesions were more suitable for PCR analysis of *V. vulnificus* infection than blood samples. We found that skin lesions were more suitable for PCR analysis of *V. vulnificus* infection than blood samples in patients treated with antibiotics before admission [15]. Studies have shown that the number of DNA copies of *V. vulnificus* in skin lesions is higher than that in blood samples and is less affected by antimicrobial therapy [15]. Further analysis showed that the relative abundance of *V. vulnificus* in the secretion of skin infection was 89.88%, and the total coverage rate was 26.02%, which was significantly higher than the relative abundance of blood samples of 52.07% and the total coverage rate of 4.80%, which further indicated that skin secretions were more sensitive to mNGS detection. The blood taken from the patient and the secretion of the infected focus were cultured on the day of admission, and no bacterial growth was found a few days later, but mNGS quickly suggested *V. vulnificus* infection, helping clinicians to adopt targeted anti-infective strategies in time, and finally recovered and discharged from hospital only after medical treatment, so as to avoid the risk of surgical debridement or even amputation. It is worth noting that the blood and secretion culture of this patient are negative, which suggests that mNGS may have higher sensitivity and specificity in the diagnosis of *V. vulnificus* infection than traditional culture methods, especially in clinical work; there are many patients who use antibiotics before admission.

Timely use of appropriate antimicrobials is the key to obtain the best prognosis. It has been proved that quinolones alone or tetracycline combined with third-generation cephalosporins have the lowest mortality and should be the first choice for *V. vulnificus* infection in ref. [17]. However, *V. vulnificus* infections may become increasingly difficult to treat as inappropriate use of antibiotics increases drug resistance. Therefore, antimicrobial agents should be customized as first-line antimicrobial agents in different countries according to the recommended treatment and reported drug resistance. In our case, patients received imipenem cilastatin combined with linezolid during hospitalization. On the second day after admission, mNGS reported *V. vulnificus* infection, considering that the data of antibacterial activity of imipenem cilastatin against *V. vulnificus* were insufficient, while the third-generation cephalosporins represented by cefoperazone sulbactam showed good antibacterial activity *in vitro*. In addition, the patients are old and have chronic kidney disease, and quinolones may increase the risk of renal injury. To effectively improve the prognosis, we decided to use the combined regimen of cefoperazone sulbactam and omocycline. Due to our early effective treatment, this patient did not undergo aggressive surgical debridement or amputation.

Therefore, for patients suspected of *V. vulnificus* infection, mNGS detection should be carried out as soon as possible to identify responsible pathogens, facilitate early adoption of targeted anti-infection strategies, early diagnosis, and early treatment, reduce disability and mortality, and improve patient prognosis.
